# The German ONKOPEDIA Guideline for Myelofibrosis in 2025—Recommendations of an MPN Expert Panel of the German Society for Hematology and Oncology (DGHO)

**DOI:** 10.1002/ijc.70595

**Published:** 2026-06-21

**Authors:** Martin Griesshammer, Haifa Kathrin Al‐Ali, Gabriela M. Baerlocher, Konstanze Döhner, Steffen Koschmieder, Nicolaus Kröger, Petro E. Petrides, Dominik Wolf, Florian H. Heidel

**Affiliations:** ^1^ University Clinic for Hematology, Oncology, Haemostaseology and Palliative Care, Johannes Wesling Medical Center Minden University of Bochum Minden Germany; ^2^ Krukenberg Cancer Center University Hospital of Halle Halle Germany; ^3^ Clinic for Hematology and Oncology Hirslanden Zürich AG Zürich Switzerland; ^4^ Medica Medical Laboratories and Pathology Medizinische Laboratorien Dr. F. Kaeppeli AG Zürich Switzerland; ^5^ Department of Internal Medicine III University Hospital Ulm Ulm Germany; ^6^ Department of Hematology, Oncology, Hemostaseology, and Stem Cell Transplantation, Faculty of Medicine RWTH Aachen University Aachen Germany; ^7^ Center for Integrated Oncology Aachen Bonn Cologne Düsseldorf (CIO ABCD) Aachen Germany; ^8^ University Clinic for Hematology and Stem Cell Transplantation Essen Germany; ^9^ Hematology Oncology Center Munich and Ludwig Maximilians University Munich Munich Germany; ^10^ Universitätsklinik für Innere Medizin V Innsbruck Austria; ^11^ Hematology, Hemostasis, Oncology, and Cell Therapy Hannover Medical School Hannover Germany

**Keywords:** guidelines, Myelofibrosis, recommendation

## Abstract

The recently published ONKOPEDIA guideline on myelofibrosis, issued under the auspices of the German Society of Hematology and Oncology (DGHO), provides an updated, evidence‐based framework for the diagnosis and management of this rare, chronic myeloproliferative neoplasm. Developed by a panel of experts (including Germany, Austria and Switzerland) nominated by the DGHO, the guideline reflects a structured and consensus‐oriented process in which internationally recognized specialists in hematology critically reviewed the available evidence, revised the written draft, and engaged in multiple rounds of discussion to ensure consistency, clinical relevance, and scientific rigor. This update builds upon the foundation of the previously available ONKOPEDIA guideline (accessible at www.onkopedia.com), but extends and refines the recommendations in light of several important recent developments.

## Graphical abstract

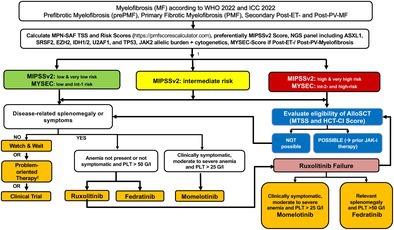

**Abbreviations**
alloSCT: allogeneic stem cell transplantation
AML: acute myeloid leukemia
DIPSS: Dynamic International Prognostic Scoring System
ELN: European LeukemiaNet
ESA: erythropoiesis‐stimulating agent
ICC: International Consensus Classification
IPSS: International Prognostic Scoring System
JAKi: Janus kinase inhibitor
MF: myelofibrosis
MIPSSv2: Mutation‐Enhanced International Prognostic Scoring System Version 2.0
MPN: myeloproliferative neoplasm
MPN‐SAF TSS: Myeloproliferative Neoplasm Symptom Assessment Form Total Symptom Score
MYSEC‐PM: myelofibrosis secondary to PV and ET prognostic model
NCCN: National Comprehensive Cancer Network
NGS: next‐generation sequencing
PMF: primary myelofibrosis
pre‐PMF: prefibrotic primary myelofibrosis
RR6: Ruxolitinib response at 6 months

## 1 Guideline Generation by an International Expert Panel

The recently published ONKOPEDIA guideline on myelofibrosis (MF), issued under the auspices of the German Society of Hematology and Oncology (DGHO), provides an updated, evidence‐based framework for the diagnosis and management of this rare, chronic myeloproliferative neoplasm (MPN). Developed by a panel of experts (including Germany, Austria and Switzerland) nominated by the DGHO, the guideline reflects a structured and consensus‐oriented process in which internationally recognized specialists in hematology critically reviewed the available evidence, revised the written draft, and engaged in multiple rounds of discussion to ensure consistency, clinical relevance, and scientific rigor. This update builds upon the foundation of the previously available ONKOPEDIA guideline (accessible at www.onkopedia.com), but extends and refines the recommendations in light of several important recent developments. These include the revised diagnostic criteria defined by the 2022 World Health Organization (WHO) and International Consensus Classification (ICC), which more clearly delineate prefibrotic primary myelofibrosis, overt fibrotic disease, and secondary MF; the growing role of cytogenetic and molecular markers in risk stratification and therapeutic decision‐making; and the approval of novel therapeutic agents, most prominently new JAK inhibitors such as fedratinib and momelotinib, which expand treatment options particularly for patients with symptomatic anemia or ruxolitinib resistance. For therapeutic decision‐making, “symptomatic anemia” should refer to anemia associated with clinically relevant manifestations such as fatigue, dyspnea on exertion, reduced exercise tolerance, dizziness, tachycardia, or transfusion requirement, while recognizing that the adverse prognostic impact of anemia in MF is independent of subjective symptom burden. Furthermore, recent advances in prognostic scoring systems and in transplantation strategies, supported by updated European LeukemiaNet (ELN) and European Society for Blood and Marrow Transplantation (EBMT) recommendations, have been incorporated into this revision. Through this iterative and collaborative process, the updated ONKOPEDIA guideline seeks to provide clinicians with a comprehensive, state‐of‐the‐art resource that integrates evolving scientific knowledge with practical guidance for patient care, thereby aligning German practice with international standards while maintaining national consensus.

## 2 The 2025 Update of the ONKOPEDIA Guidelines on MF

In comparison to the previous version, the 2025 ONKOPEDIA guideline on myelofibrosis introduces several important updates that reflect recent scientific and clinical advances and are directly relevant for daily practice. Key innovations include:

- Stronger emphasis on symptom burden: The Myeloproliferative Neoplasm Symptom Assessment Form (MPN‐SAF TSS) is now recognized as an equally weighted parameter alongside hematologic and clinical findings, underscoring the central role of patient‐reported outcomes in disease monitoring and therapeutic decision‐making.
- Diagnosis and risk stratification: Updated definitions according to the 2022 WHO and ICC classifications refine the distinction between prefibrotic and overt MF.
- Molecularly informed risk scores such as MIPSSv2 and MYSEC have been integrated, together with systematic use of next‐generation sequencing (NGS) and cytogenetics, to achieve more precise prognostication.
- Therapeutic principles: JAK inhibitors (ruxolitinib, fedratinib, and momelotinib) are established as standard of care for symptomatic disease, with therapeutic choice guided by risk profile, anemia status, and splenomegaly.
- New therapeutic options: Combination approaches, such as the use of JAK inhibitors together with erythropoiesis‐stimulating agents and investigational strategies including luspatercept for anemia, are discussed. Allogeneic stem cell transplantation remains the only curative treatment modality and is emphasized for patients with high‐risk disease.
- Clinical practice tools: Early identification of ruxolitinib treatment failure, particularly through the Ruxolitinib Response after 6 months (RR6) score, is highlighted as critical for timely treatment adaptation and for referral to transplantation evaluation when appropriate.

### 2.1 Stronger Emphasis on Symptom Burden

The 2025 ONKOPEDIA guideline emphasizes the central role of patient‐reported symptom burden in the diagnosis, monitoring, and therapeutic decision‐making of MF. Historically, hematologic parameters such as hemoglobin levels, leukocyte counts, and splenomegaly dominated risk assessment and treatment algorithms. However, emerging evidence demonstrates that symptom severity not only correlates with impaired quality of life [1, 2, 3] but also with overall survival [4, 5]. Consequently, the guidelines now elevate symptoms to an equivalent status alongside clinical and hematological parameters. To standardize symptom evaluation, the use of validated instruments such as the MPN‐SAF TSS [6] is recommended. This tool captures 10 core MF‐associated symptoms—including fatigue, night sweats, bone pain, pruritus, anorexia, weight loss, and splenomegaly‐related discomfort—by grading their frequency and severity on a numerical scale. The resulting total score provides an objective and reproducible measure of symptom burden. A reduction of ≥ 50% in MPN‐SAF TSS is widely accepted as a clinically meaningful treatment response, according to consensus from the International Working Group for Myeloproliferative Neoplasms Research and Treatment (IWG‐MRT). Importantly, the guidelines stress the practical implementation of symptom assessment at every clinical encounter, both to track disease evolution and to evaluate therapeutic efficacy. Symptom progression, even in the absence of overt hematologic progression, is now regarded as a sufficient indication for therapeutic escalation or modification. This paradigm shift ensures that patient‐reported outcomes are systematically incorporated into decision‐making, reinforcing a patient‐centered approach. Ultimately, by weighting symptom burden on par with traditional clinical endpoints, the updated guideline acknowledges the multidimensional nature of MF and aligns clinical practice with the dual objectives of prolonging survival and improving quality of life.

### 2.2 Recommendations for the Pre‐Fibrotic Phase of Myelofibrosis

The pre‐fibrotic phase of primary myelofibrosis (pre‐PMF) is now firmly recognized as a distinct clinical and pathological entity within the MPN spectrum, clearly separated from essential thrombocythemia (ET) and overt PMF by the WHO (2016, 2022) and ICC (2022) classifications [7, 8]. The differences between WHO 2022 and ICC 2022 for pre‐PMF are mainly related to wording, emphasis, and the presentation of clonality criteria, whereas the overall diagnostic framework remains highly concordant. Accurate diagnosis is essential but remains challenging, as clinical overlap with ET and early PMF is frequent, and misclassification may lead to inappropriate treatment strategies. Diagnosis requires integration of bone marrow histopathology—characterized by hypercellularity, atypical megakaryopoiesis, and minimal reticulin fibrosis (grade 0–1)—with molecular findings (*JAK2*, *CALR*, or *MPL* mutations, or other clonal markers) and minor criteria including anemia, leukocytosis, splenomegaly, or elevated LDH. Communication between clinicians and expert hematopathologists is strongly recommended to ensure diagnostic precision.

Clinically, pre‐PMF is heterogeneous [9, 10]. Some patients remain asymptomatic, while others present with constitutional symptoms, cytopenias, or increased thromboembolic risk. Importantly, patients with pre‐PMF have a worse prognosis than ET, with higher rates of progression to overt MF, secondary acute myeloid leukemia (AML), and inferior overall survival. Subgroups, such as those with cytopenic pre‐PMF or high‐risk molecular mutations (e.g., *ASXL1, SRSF2, EZH2, IDH1/2, TP53*), demonstrate particularly poor outcomes, emphasizing the role of molecular profiling in risk assessment. Traditional risk scores (IPSS, DIPSS) can be applied, but molecularly informed models (MIPSSv2, GIPSS) may better reflect the biology of pre‐PMF.

Treatment recommendations are risk‐adapted and mirror therapeutic strategies used in ET and overt PMF. Low‐risk, asymptomatic patients can often be managed with observation and low‐dose aspirin to reduce thrombotic events. Cytoreductive therapy with hydroxyurea or interferon‐alpha is indicated in symptomatic patients, or in those with high counts or thrombotic risk. Interferon, particularly ropeginterferon‐alpha, is emerging as a preferred disease‐modifying therapy in younger patients with long life expectancy, supported by early data suggesting reduction in JAK2 allele burden and potential delay of disease progression. JAK inhibitors such as ruxolitinib may be considered in selected symptomatic pre‐PMF patients with splenomegaly or systemic symptoms, though prospective data specific to this subgroup are lacking, and their role remains under investigation. Importantly, the guidelines emphasize that pre‐PMF is not invariably progressive, but younger patients and those with molecular high‐risk features require close monitoring, with allogeneic hematopoietic stem cell transplantation considered in selected cases. Management of thrombotic events in PMF should be individualized according to thrombotic site, bleeding risk, platelet count, and concomitant cytopenias. In general, anticoagulation is the backbone of therapy for venous thromboembolism, including thrombosis at atypical sites, whereas antiplatelet therapy may be considered mainly in selected arterial or microvascular manifestations. In contrast to ET or PV, thrombotic management in PMF is more frequently complicated by anemia, thrombocytopenia, advanced splenomegaly, portal hypertension, and an increased bleeding risk; therefore, treatment decisions require careful case‐by‐case evaluation.

Overall, the updated guideline recommendations for pre‐PMF highlight the importance of precise diagnosis, structured symptom assessment, molecularly informed risk stratification, and tailored therapy. They underscore the need for vigilance in identifying high‐risk phenotypes and for integrating evolving therapeutic approaches to optimize outcomes in this early but clinically significant phase of MF. The clinical heterogeneity of pre‐PMF remains a major challenge, and prospective disease‐specific clinical trials are limited. As a consequence, current therapeutic recommendations are largely extrapolated from ET and overt PMF, underscoring the need for dedicated prospective studies in this subgroup.

### 2.3 Use of Molecular Risk Scores

Prognostication in MF has evolved from purely clinical scores to integrated molecular models. The 2025 ONKOPEDIA guideline incorporates WHO/ICC 2022 criteria and NGS‐based risk assessment, reflecting the growing importance of cytogenetic and mutational data. While traditional systems such as IPSS and DIPSS remain useful, their clinical focus limits precision. MIPSS70+ v2.0 (MIPSSv2) is recommended as the preferred model for primary MF, combining clinical variables with high‐risk mutations and cytogenetics to stratify patients into five risk categories with defined survival estimates. For secondary MF, MYSEC‐PM provides a validated alternative. Both scores allow dynamic reassessment and are easily applied in clinical practice via online tools. These models directly inform management: low‐risk patients are typically observed or treated symptomatically, whereas high‐ and very‐high‐risk patients should be considered early for allogeneic stem cell transplantation [11, 12]. In secondary MF, recently updated versions of the MYSEC model incorporating molecular and cytogenetic variables may further refine prognostic assessment and should be considered in centers where these data are available [13]. For transplant decision‐making, MIPSS70+ v2.0 is particularly relevant in primary MF, while MYSEC‐PM should additionally be considered in secondary MF. The guideline also stresses the importance of comprehensive NGS and cytogenetic testing at baseline and during follow‐up, particularly in transplant‐eligible individuals, to refine risk stratification and to anticipate progression and clonal evolution [14]. By embedding molecularly informed scores into clinical algorithms, the 2025 guideline ensures that therapeutic decisions reflect the underlying disease biology as well as the clinical phenotype.

### 2.4 Update on JAK‐Inhibitor Use and Stratification

The therapeutic landscape of MF has been transformed by the advent and refinement of JAK‐inhibitor therapy. The 2025 ONKOPEDIA guideline designates JAK inhibitors as the standard of care for symptomatic MF, targeting splenomegaly and constitutional symptoms. Ruxolitinib remains the first‐line agent for the majority of patients, supported by extensive evidence demonstrating improvements in symptom control and overall survival. However, recent approvals expand the therapeutic repertoire. Fedratinib, a selective JAK2/FLT3 inhibitor, provides an important alternative in patients intolerant to or progressing under ruxolitinib. Although fedratinib is approved for use in the front‐line setting, ONKOPEDIA positions it primarily as a second‐line option, reflecting clinical practice patterns and comparative experience. However, it may be considered as a first‐line alternative in selected patients, such as those with contraindications to ruxolitinib or specific comorbidities, such as non‐melanoma skin cancer, lymphoproliferative disorders, or thrombocytopenia. More recently, momelotinib, a JAK1/2 and ACVR1/ALK2 inhibitor, has received European approval for MF patients with moderate to severe anemia, including those previously treated with ruxolitinib. Unlike other JAK inhibitors, momelotinib improves anemia outcomes, reducing transfusion dependence, which is of particular relevance in advanced disease stages [15, 16, 17]. Therapeutic choice should therefore be stratified according to the patient's clinical profile: ruxolitinib for classic symptomatic disease with splenomegaly, fedratinib for ruxolitinib failure, and momelotinib in the presence of anemia. Initial dosing of ruxolitinib should primarily follow the approved platelet‐based prescribing information. In patients with marked baseline anemia, caution is warranted, but systematic underdosing should be avoided; in appropriate patients with clinically relevant anemia, momelotinib may represent a preferable first‐line JAK inhibitor option. Although momelotinib offers clinically meaningful benefits in anemia control, uncertainties remain regarding its long‐term comparative efficacy and survival benefit relative to ruxolitinib or fedratinib, particularly outside the context of clinical trials. The guidelines further highlight the role of combination strategies, such as ruxolitinib or momelotinib with erythropoiesis‐stimulating agents [18, 19, 20], which may provide synergistic benefit in selected patients. It should be noted that many combination approaches, including JAK inhibitor–based combinations with ESAs or luspatercept, are supported predominantly by early‐phase or non‐randomized studies. Robust comparative data from randomized trials are still lacking. Importantly, treatment selection must always be contextualized within the broader risk profile determined by molecular and clinical scores. JAK inhibitors are not curative, but their capacity to improve symptom burden, performance status, and splenomegaly is crucial not only for long‐term palliation but also as a bridge to transplantation in eligible patients. Accordingly, the guidelines recommend systematic evaluation of JAK‐inhibitor response, stratified according to anemia severity, platelet counts, and splenomegaly. This nuanced stratification ensures optimal agent selection, minimizes toxicity, and maximizes both quality of life and survival benefit for patients with MF.

### 2.5 New Therapeutic Options

Beyond the established JAK‐inhibitor backbone, the 2025 ONKOPEDIA guideline highlights several emerging therapeutic strategies that may soon expand treatment possibilities. Combination regimens represent a major area of development. Clinical trials investigating the co‐administration of JAK inhibitors with erythropoiesis‐stimulating agents have demonstrated improved anemia responses, with synergistic effects observed particularly when momelotinib is combined with erythropoietin. Prospective controlled data on danazol in MF may also be informed by the danazol comparator arm of the MOMENTUM trial, which provides contemporary data on efficacy and safety in anemic patients. Another promising approach is the use of luspatercept, a TGF‐β superfamily ligand trap that promotes erythroid maturation. Early‐phase trials (e.g., ACE‐536‐MF‐001, ODYSSEY) indicate that luspatercept, alone or in combination with JAK inhibitors [21], may alleviate anemia and reduce transfusion burden in MF patients. While not yet part of routine clinical care, these strategies offer hope for addressing the unmet need of anemia management, a major driver of morbidity and mortality in MF. Additional investigational avenues include novel JAK inhibitors, BET inhibitors, telomerase inhibitors, and agents targeting the bone marrow microenvironment, reflecting a broader shift toward combination and pathway‐directed therapy [22]. Despite these advances, the guidelines reaffirm that allogeneic stem cell transplantation remains the only curative option. The integration of new agents is thus envisioned as a means to improve symptom control, reduce disease burden, and optimize pre‐transplant conditioning. By presenting these therapeutic innovations alongside established standards, the guideline underscores the dynamic evolution of MF management, balancing evidence‐based care with forward‐looking strategies. Patients should be considered for clinical trial enrollment whenever possible, both to access these novel agents and to contribute to the rapidly expanding body of evidence guiding future practice.

### 2.6 Identification of Ruxolitinib Failure by Clinical Practice Tools

The recognition of treatment failure under ruxolitinib is a crucial determinant of patient outcome, as delayed intervention can compromise survival and preclude timely transition to alternative therapies or transplantation. The 2025 ONKOPEDIA guideline introduces structured clinical tools to standardize this assessment, with particular emphasis on the “RR6” score. This model evaluates treatment efficacy based on three parameters: spleen response (measured by volume reduction or palpable splenomegaly), symptom burden, and hematologic parameters (notably hemoglobin and platelet counts). Patients who fail to achieve adequate improvements across these domains at 6 months are classified as “RR6 high‐risk,” indicating suboptimal response [23]. Such patients have markedly poorer long‐term outcomes if therapy is continued without modification. The guideline therefore recommends early identification of RR6 high‐risk patients to prompt timely therapeutic adaptations, including switching to an alternative JAK inhibitor, referral for clinical trial participation, or expedited evaluation for allogeneic transplantation in eligible individuals. This proactive approach contrasts with historical reliance on delayed recognition of treatment resistance, which often resulted in progressive disease and loss of transplant eligibility. Importantly, the RR6 model is validated by large prospective cohorts that confirmed its prognostic value. In routine practice, application of RR6 should be coupled with structured symptom assessments (MPN‐SAF TSS) and dynamic risk scores (MIPSSv2 or MYSEC), ensuring a comprehensive evaluation of disease trajectory. In patients with intermediate‐1 risk disease, the iRR6 model may provide additional granularity for early response assessment during ruxolitinib therapy and may support earlier therapeutic reassessment in selected patients [24]. Together, these tools empower clinicians to identify ruxolitinib non‐responders early, individualize therapeutic planning, and preserve curative opportunities. By embedding the RR6 model into routine care, the 2025 guideline provides a pragmatic framework for optimizing outcomes in patients receiving JAK inhibitors. For combined hydroxyurea and ruxolitinib therapy, additional support is provided by larger published real‐world cohorts, which may better reflect feasibility and tolerability in routine clinical practice [25]. While the RR6 model represents a valuable and clinically validated tool for early identification of suboptimal responders, it has several limitations. Its reliance on spleen volume measurements may restrict applicability in settings where standardized imaging is not routinely available, and it may misclassify patients with delayed but ultimately meaningful responses. Therefore, RR6 should be interpreted in the context of longitudinal clinical assessment.

## 3 The Updated MF‐ONKOPEDIA‐Guidelines in Context of International Treatment Recommendations: Comparison With the NCCN‐Guidelines for MF

### 3.1 Risk Stratification and Diagnostic Frameworks

Both ONKOPEDIA and NCCN base MF diagnosis on the 2022 WHO/ICC classifications, but they differ in how prognostic models are prioritized (Table 1). The National Comprehensive Cancer Network (NCCN) guidelines, widely used in the United States, serve as an important international reference framework for comparison due to their comprehensive and regularly updated evidence‐based recommendations. NCCN presents parallel options for PMF, including DIPSS/DIPSS‐Plus and mutation‐informed scores such as MIPSS‐70 and MIPSS‐70+ v2.0, while reserving MYSEC‐PM for post‐PV/ET MF. ONKOPEDIA also integrates molecular risk assessment but applies a clearer hierarchy: MIPSSv2 is preferred for PMF, and MYSEC for secondary MF, supported by a dedicated calculator and routine NGS plus cytogenetics. In contrast to NCCN's more pluralistic approach, ONKOPEDIA provides a more defined default pathway, with stronger emphasis on comprehensive baseline molecular profiling. It also explicitly notes that FISH is not a substitute for conventional karyotyping in prognostic scoring, particularly for MIPSSv2. As a result, both frameworks may place patients into similar risk categories, but ONKOPEDIA more strongly promotes genome‐informed risk assessment in routine practice, whereas NCCN maintains broader model flexibility and highlights referral to specialized centers.

**TABLE 1:** MF‐ONKOPEDIA‐guidelines in context of international treatment recommendations: Comparison with the NCCN‐guidelines for myelofibrosis (MF).

Domain	ONKOPEDIA (2025)	NCCN (v1.2026)	Main practical distinction
Risk models/prognostic framework	Prioritizes a molecularly informed hierarchy: MIPSS70+ v2.0 (MIPSSv2) is favored for PMF; MYSEC‐PM is used for secondary MF. Strong emphasis is placed on baseline NGS plus cytogenetics, especially in transplant‐eligible patients; the guideline explicitly states that molecular/cytogenetic work‐up should help identify transplant candidates early. If cytogenetics are unavailable, MIPSS‐70 can still be used.	Uses several accepted models in parallel: for PMF, MIPSS‐70 or MIPSS‐70+ v2.0 are preferred, with DIPSS or DIPSS‐Plus as alternatives depending on data availability; for post‐PV/post‐ET MF, MIPSS‐70+ v2.0, DIPSS‐Plus, and MYSEC‐PM are listed. NCCN also recommends multigene molecular testing and bone marrow cytogenetics for diagnosis and prognostic stratification.	Both frameworks are now molecularly informed, but ONKOPEDIA is more prescriptive in making MIPSSv2 the default biologically driven score, whereas NCCN is more pluralistic and allows several validated models to coexist.
JAK inhibitor strategy	Centers treatment around ruxolitinib, fedratinib, and momelotinib, reflecting EMA/German practice. Momelotinib is highlighted particularly for patients with clinically relevant anemia. Pacritinib is discussed, but remains not routinely available/off‐label in Germany. After ruxolitinib failure, ONKOPEDIA‐associated German practice steers mainly toward fedratinib or momelotinib. ONKOPEDIA also uses RR6 more explicitly to define inadequate ruxolitinib response and trigger treatment change.	Offers a broader FDA‐aligned spectrum: ruxolitinib and fedratinib are category 1 options for higher‐risk MF with platelets ≥ 50 × 10^9/L; pacritinib is a category 1 preferred option for higher‐risk MF with platelets < 50 × 10^9/L when transplant is not feasible; momelotinib is incorporated across symptom/anemia settings. RR6 is referenced as a useful assessment tool, but application is left more to clinician discretion.	The largest divergence is pacritinib: it is structurally embedded in NCCN, especially for profound thrombocytopenia, but is not part of routine ONKOPEDIA/German care pathways. ONKOPEDIA also appears more prescriptive regarding RR6‐driven switching after ruxolitinib failure.
Anemia management	Emphasizes treatment of MF‐associated anemia within a broader problem‐oriented strategy. Momelotinib is highlighted because of its favorable anemia/transfusion profile. Erythropoietin may be considered when serum EPO is low, with ONKOPEDIA citing a favorable response particularly when serum EPO < 125 U/L; combination with ruxolitinib is discussed but not broadly endorsed because of limited data. Luspatercept is presented mainly as an investigational/off‐label or combinatorial option rather than routine standard care.	Has a dedicated MF‐3 anemia pathway. Momelotinib is listed as a preferred regimen; depending on spleen/symptom status, options include pacritinib, danazol, luspatercept, and ESAs if serum EPO < 500 mU/mL, either alone or added to an ongoing JAK inhibitor. NCCN explicitly permits JAK inhibitor combinations for persistent anemia when splenomegaly/symptoms are otherwise controlled.	Both recognize the special role of momelotinib in anemic MF. However, NCCN formalizes anemia management more explicitly and uses a broader ESA threshold (EPO < 500 mU/mL), while ONKOPEDIA is more cautious, with ESAs and luspatercept framed more selectively and often in a trial−/case‐based context.
Transplant indications/timing	Strongly aligned with EBMT/ELN‐style early referral. Allogeneic transplantation remains the only curative option and is generally indicated in transplant‐fit patients with high or very high risk by MIPSS70+ v2.0; ONKOPEDIA also stresses early identification of candidates through molecular/cytogenetic profiling and mentions biological age up to about 75 years. In patients on JAK inhibitors, RR6 assessment after 6 months is used to identify those needing prompt transplant evaluation. Pre‐transplant JAK inhibitor use is emphasized in symptomatic splenomegaly.	Also states that allogeneic HCT is the only curative option, but specifies referral and recommendation thresholds somewhat differently: referral to HCT experts is advised for DIPSS‐Plus Int‐1 or MIPSS intermediate or higher disease, while transplant is recommended for DIPSS‐Plus/MYSEC Int‐2 or high‐risk and MIPSS70/MIPSS‐70+ high‐risk disease. NCCN also incorporates MTSS for post‐transplant risk estimation and details pre‐transplant JAK inhibitor considerations.	Both endorse early transplant thinking, but ONKOPEDIA pushes earlier, biology‐driven referral, especially through MIPSSv2 and RR6‐based reassessment, whereas NCCN defines transplant referral and transplant recommendation with more score‐specific thresholds across several models.

### 3.2 Weighting of Symptom Burden in Initial Assessment and Follow‐Up

Both guidelines recommend routine symptom assessment with the MPN‐SAF TSS (MPN‐10), but ONKOPEDIA gives symptoms a more central role in decision‐making. It treats symptom burden as equal to laboratory and clinical findings and allows treatment escalation or switch based on symptom progression alone. NCCN also recommends baseline and serial TSS assessment, but uses these scores more as guidance within clinician judgment, noting that even less than 50% improvement may still support continued JAK inhibitor therapy. As a result, ONKOPEDIA may encourage earlier symptom‐driven treatment changes, including transplant evaluation, whereas NCCN more often places symptom assessment within broader risk‐adapted management pathways.

### 3.3 JAK‐Inhibitor Selection and Sequencing: Platelets, Anemia, and Geography

NCCN offers a broad, FDA‐aligned armamentarium reflecting US regulatory approvals. For higher‐risk MF with platelets ≥ 50 × 10^9/L, ruxolitinib or fedratinib are category 1 options; if platelets < 50 × 10^9/L and transplant is not feasible, pacritinib is a category 1 preferred regimen, with momelotinib listed (category 2B). In contrast, ONKOPEDIA structures choices around EU availability and payer guidance, recognizing ruxolitinib and fedratinib while integrating the 2023–2024 European/Swiss approvals and real‐world data for momelotinib, particularly in anemic MF (Table 1). Crucially, a late‐2024 joint statement by German authorities (BfArM/MDK Oncology Competence Center) directs second‐line JAK inhibition after ruxolitinib to a binary decision between fedratinib and momelotinib, with “all other combinations deemed off‐label”—implicitly excluding pacritinib from routine use in Germany. Accordingly, while NCCN can preferentially deploy pacritinib for severe thrombocytopenia and positions momelotinib variably (including for anemia), ONKOPEDIA channels European clinicians toward fedratinib or momelotinib in second line and leverages momelotinib's anemia profile earlier, reflecting regulatory and reimbursement realities rather than differences in pathobiology.

### 3.4 Management of MF‐Associated Anemia and “New” Options

NCCN addresses anemia in a dedicated MF‐3 pathway and, outside clinical trials, lists momelotinib as a preferred option, with pacritinib, ESAs, danazol, and luspatercept used alone or in combination depending on anemia, spleen, and symptom control. It also explicitly supports adding ESAs or luspatercept to ongoing JAK inhibitor therapy when splenomegaly is controlled but anemia persists. ONKOPEDIA likewise recognizes momelotinib's particular value for anemia and transfusion dependence and includes luspatercept as an emerging combination strategy, while retaining hydroxyurea for hyperproliferative disease. Thus, both guidelines acknowledge momelotinib's anemia‐directed role, but differ in implementation. NCCN more formally incorporates pacritinib and combination approaches into routine management, whereas ONKOPEDIA reflects EMA approval status and German practice, where add‐on strategies are used more selectively and often case by case.

### 3.5 Recognition of Ruxolitinib Failure and Timing of Transition

Both guidelines acknowledge the need for early identification of suboptimal JAK response, but ONKOPEDIA operationalizes the RR6 model more assertively. The German text details the RR6 inputs (ruxolitinib dose < 20 mg bid, ≤ 30% palpable spleen reduction at 3/6 months, and red‐cell transfusion need) and stratifies 6‐month outcomes into low/intermediate/high risk to trigger timely second‐line switch and transplant planning. NCCN references RR6 in MF‐1/MF‐2 footnotes (“use RR6 to assess ruxolitinib”) and recommends response‐ and symptom‐driven continuation or change, but leaves the decision “at the discretion of the clinician”. Thus, ONKOPEDIA provides a prescriptive, time‐boxed checkpoint at 6 months to avoid therapeutic inertia, harmonizing with its stronger symptom co‐primacy; NCCN embeds RR6 as an adjunct within broader monitoring guidance. Together with NCCN's category‐1 endorsement of pacritinib for platelets < 50 × 10^9/L, these differences shape divergent next steps after RR6‐defined nonresponse: ONKOPEDIA channels toward fedratinib or momelotinib (plus transplant referral), while NCCN may favor pacritinib in profoundly thrombocytopenic patients or momelotinib when anemia predominates.

### 3.6 Transplant Referral and Timing Strategies

Both guidelines recognize allogeneic stem cell transplantation as the only potentially curative approach, but they differ in referral urgency and timing frameworks. NCCN recommends referral to HCT experts for all patients with DIPSS‐Plus Int‐1 or MIPSS‐intermediate or higher risk, with transplant recommended for DIPSS‐Plus/MYSEC Int‐2/high‐risk or MIPSS70/MIPSS‐70+ high‐risk disease. The guidelines incorporate the Myelofibrosis Transplant Scoring System (MTSS) [26] for post‐transplant survival prediction and emphasize patient selection based on age, performance status, comorbidities, psychosocial factors, and caregiver availability. ONKOPEDIA adopts the 2023 EBMT/ELN recommendations more directly, advocating early transplant referral and incorporating biological age considerations up to approximately 75 years. The German guideline specifically mandates that transplant‐eligible patients receiving JAK inhibitors undergo RR6‐based evaluation after 6 months, with high‐risk scores prompting immediate transplant evaluation. Additionally, ONKOPEDIA emphasizes that patients with splenomegaly ≥ 5 cm or splenomegaly‐related symptoms should receive JAK inhibitor therapy prior to transplant, and details peri‐transplant JAK inhibitor management protocols based on recent evidence showing improved engraftment and reduced GVHD. Splenic irradiation and splenectomy should both be regarded as individualized options in selected patients with marked splenomegaly, particularly in the transplant setting. Splenic irradiation may be considered as a bridge to transplantation and is generally less invasive, whereas splenectomy may offer a more substantial reduction of splenic burden in selected cases but is associated with considerable perioperative morbidity and mortality. As comparative prospective data are lacking, the choice between both strategies should be made on a case‐by‐case basis according to symptom burden, spleen size, cytopenias, transplant timing, and institutional expertise [27].

### 3.7 Novel Therapeutic Approaches and Pipeline Integration

NCCN maintains a conservative stance toward investigational agents, consistently recommending clinical trial enrollment as preferred options while providing structured pathways for approved agents. The guidelines mention luspatercept, ESAs, and combination approaches within established treatment algorithms but emphasize proven therapies over experimental strategies. ONKOPEDIA, conversely, dedicates substantial attention to emerging therapeutics and pipeline developments, including detailed discussions of imetelstat, TGF‐β pathway inhibitors (luspatercept, elritercept), BET inhibitors (pelabresib), and innovative CALR‐targeted approaches (blocking antibodies, bispecific T‐cell redirectors, vaccines, CAR‐T cells) [28]. The German guideline's approach reflects a more research‐intensive environment where clinicians are expected to be familiar with cutting‐edge developments and consider patients for novel therapeutic approaches. This difference may stem from the European academic medical system's emphasis on translational research integration and the ONKOPEDIA platform's role in bridging clinical practice with emerging science. While both guidelines advocate for clinical trial participation, ONKOPEDIA provides more granular detail about specific investigational strategies, potentially facilitating earlier adoption of promising therapies in appropriate clinical contexts.

## 4 Summary

Taken together, the 2025 ONKOPEDIA guideline on MF represents a comprehensive update that integrates recent advances in disease classification, risk stratification, and therapeutic management, while maintaining a strong patient‐centered perspective. Central to this revision is the recognition that symptom burden, as measured by validated tools such as the MPN‐SAF TSS, carries prognostic significance and must be considered equally alongside clinical and hematologic parameters. The guideline also reinforces the importance of molecularly informed scoring systems, ensuring that therapeutic decisions increasingly reflect underlying disease biology rather than clinical presentation alone. JAK inhibitors remain the therapeutic backbone, with ruxolitinib, fedratinib, and momelotinib providing complementary options depending on clinical context, particularly in patients with anemia or after ruxolitinib failure. Importantly, the systematic use of the RR6 score at 6 months is recommended to avoid treatment inertia and to support timely adjustments or referral for transplantation. Pre‐fibrotic PMF is acknowledged as a distinct disease phase requiring careful diagnosis and tailored, risk‐adapted therapy, while investigational strategies such as combination regimens or anemia‐targeted agents illustrate the dynamic evolution of the treatment landscape. In comparison to international recommendations such as the NCCN guidelines, ONKOPEDIA places stronger emphasis on molecular testing, symptom‐led decision‐making, and early transplant referral, while reflecting European regulatory and reimbursement frameworks in its therapeutic algorithms (Table 1). Taken together, the updated guideline underscores the necessity of integrating biological, clinical, and patient‐reported data into management decisions. It provides a clear and practical framework for improving outcomes in MF, while also highlighting areas where further clinical research and harmonization across international guidelines will be critical.

## 5 Key Messages

The 2025 ONKOPEDIA guideline integrates updated WHO/ICC classifications, molecular risk models, and patient‐reported outcomes into a unified framework for MF care.

Symptom burden, assessed by the MPN‐SAF TSS, is now weighted equally with hematologic and clinical findings in therapeutic decision‐making.

JAK inhibitors remain the treatment backbone, with ruxolitinib, fedratinib, and momelotinib positioned according to clinical context; the RR6 score is recommended for early identification of treatment failure.

Pre‐fibrotic PMF is recognized as a distinct entity requiring accurate diagnosis, close monitoring, and risk‐adapted therapy, including interferon in selected younger patients.

Compared with NCCN, ONKOPEDIA emphasizes molecular testing, symptom‐driven management, and European‐specific therapeutic guidance, while underscoring the need for continued clinical research and international harmonization.

This guideline is intended to support hematologists and other treating physicians in the diagnostic work‐up, risk stratification, treatment selection, and longitudinal management of patients with primary and secondary MF in routine clinical practice.

Limitations: The recommendations presented in this guideline summary reflect the European, and specifically German, regulatory and reimbursement framework, which may limit generalizability to other healthcare systems. In addition, the strength of evidence varies across different aspects of MF management, with robust data available for JAK inhibitor therapy but more limited evidence for pre‐PMF treatment and combination strategies. Furthermore, the rapidly evolving therapeutic landscape may render certain recommendations time sensitive. Finally, access to advanced molecular diagnostics and standardized spleen volumetry may not be uniformly available across all clinical settings.

## Author Contributions

**Martin Griesshammer:** conceptualization, writing – original draft, writing – review and editing, methodology, formal analysis, supervision, data curation. **Haifa Kathrin Al‐Ali:** conceptualization, writing – review and editing, formal analysis, data curation. **Gabriela M. Baerlocher:** conceptualization, writing – review and editing, formal analysis, data curation. **Konstanze Döhner:** conceptualization, writing – review and editing, formal analysis, data curation. **Steffen Koschmieder:** conceptualization, writing – review and editing, formal analysis, data curation. **Nicolaus Kröger:** conceptualization, writing – review and editing, formal analysis, data curation. **Petro E. Petrides:** conceptualization, writing – review and editing, formal analysis, data curation. **Dominik Wolf:** conceptualization, writing – review and editing, formal analysis, data curation. **Florian H. Heidel:** conceptualization, visualization, formal analysis, data curation, writing – review and editing.

## Conflicts of Interest

**M.G**.: Consultancy and Honorarium from AOP Orphan, Novartis, BMS, AbbVie, Pfizer, Roche, Janssen, Gilead, AstraZeneca, Sierra, Lilly, GSK. **H.K.A.‐A**.: Research support: Incyte, BMS. Consultancy: BMS, Incyte, Novartis, AbbVie, AOP Pharma, Takeda, GSK, Geron, Otsuka, Stemline, MSD, Kartos, Telios. Honoraria: BMS, Novartis, AbbVie, AOP, Takeda, GSK, Otsuka, Stemline. **G.M.B**.: Consultant for GSK and Geron. **K.D**.: Consultant: BMS/Celgene, AOP, Novartis, AbbVie, GSK MSD. Research funding: Novartis, BMS/Celgene. **S.K**. reports institutional grants from Geron, Janssen, AOP Pharma, and Novartis; consulting fees from Pfizer, Incyte, Ariad, Novartis, AOP Pharma, BMS/Celgene, Geron, Janssen, CTI BioPharma, Roche, Bayer, GSK, Sierra Oncology, PharmaEssentia, Protagonist Therapeutics, Takeda, and MSD; honoraria from Novartis, BMS/Celgene, Pfizer, AstraZeneca, iOMEDICO, Takeda, and MSD; travel support from Alexion, Novartis, BMS, Incyte, AOP Pharma, CTI BioPharma, Pfizer, Celgene, Janssen, Geron, Roche, AbbVie, GSK, Sierra Oncology, Kartos, AstraZeneca, Protagonist Therapeutics, Takeda, MSD, and iOMEDICO; advisory board fees from Pfizer, Incyte, Ariad, Novartis, AOP Pharma, BMS, Celgene, Geron, Janssen, CTI BioPharma, Roche, Bayer, GSK, Sierra Oncology, PharmaEssentia, Protagonist Therapeutics, Takeda, and MSD; an issued patent with RWTH Aachen University; and unpaid leadership roles with the German MPN Study Group, DGHO, and EHA. **D.W**.: Research funding: Ariad, Pfizer, Novartis, BMS, Roche, AOP, MSD. Fee or other reimbursement: Ariad, Pfizer, Novartis, BMS, Roche, Amgen, Incyte, Lilly, Takeda, AOP. **F.H.H**. is a consultant for BMS/Celgene, AOP, Novartis, CTI, Janssen, AbbVie, GSK, Merck, Kartos, Telios, Silence, Prelude, Takeda and Sumitomo and received research funding from: Novartis, BMS/Celgene and CTI. The other authors declare no conflicts of interest.

## Data Availability

The data that support the findings of this study are available from the corresponding author upon reasonable request.
